# A Current Review of the Diagnostic and Treatment Strategies of Hepatic Encephalopathy

**DOI:** 10.1155/2012/480309

**Published:** 2012-10-21

**Authors:** Z. Poh, P. E. J. Chang

**Affiliations:** ^1^Division of Medicine, Department of Gastroenterology & Hepatology, Singapore General Hospital, Outram Road, Singapore 169608; ^2^Duke-NUS Graduate Medical School, 8 College Road, Singapore 169857

## Abstract

Hepatic encephalopathy (HE) is a serious and potentially fatal complication in patients with cirrhotic liver disease. It is a spectrum ranging from minimal hepatic encephalopathy (MHE) without recognizable clinical symptoms or signs, to overt HE with risk of cerebral edema and death. HE results in diminished quality of life and survival. The broad range of neuropsychiatric manifestations reflects the range of pathophysiological mechanisms and impairment in neurotransmission that are purported to cause HE including hyperammonemia, astrocyte swelling, intra-astrocytic glutamine, upregulation of 18-kDa translocator protein (TSPO) (formerly known as peripheral benzodiazepine receptor or PBTR), and manganese. There is a myriad of diagnostic tools including simple bedside clinical assessment, and more complex neuropsychological batteries and neurophysiological tests available today. Current treatment strategies are directed at reducing ammonia, with newer agents showing some early promise. This paper describes the pathophysiology of the disease and summarises current diagnostic and treatment therapies available.

## 1. Introduction

Hepatic encephalopathy (HE) is a common complication in patients with cirrhotic liver disease. HE not only results in a diminished quality of life, but confers a poorer prognosis in patients with underlying liver cirrhosis [1]. HE is an important event in the natural history of cirrhosis and is an independent predictor of mortality in patients with acute on chronic liver failure [2, 3]. In severe cases, it can even lead to coma or death [1]. Mortality is extremely high in overt HE with cerebral edema, and temporizing measures are often inadequate [4]. 1-year mortality for patients with severe HE in intensive care unit (ICU) is 54%, with requirement for inotropic support and acute kidney injury identified as independent predictors of ICU death and 1-year mortality [5].

Episodes of overt HE result in frequent hospitalizations, and pose a formidable burden on the healthcare system [6]. Its subclinical manifestation can be found in asmany as about 50% of cirrhotic patients without overt encephalopathy [7]. Even minimal hepatic encephalopathy (MHE), the mildest form of HE, first described by Zeegen et al. in 1970 [8], without clinically overt signs of impaired cognition has been demonstrated to diminish healthcare related quality of life [9–11] and portends a poorer prognosis [12].

Whilst MHE has been reported to occur commonly in cirrhotics, its natural history has not been well described. Its main impact appears to be cognitive and drives impairment and its attendant social implications [13, 14]. However, a local Singapore cohort study in 2009 revealed that a significant proportion of patients with well-compensated MHE can revert to a normal state [15].

Newer therapeutics and treatment strategies have emerged since the American College of Gastroenterology released their practice guidelines on HE in 2001 [16] and have offered renewed hope upon the horizon to both patients and physicians alike, for the management of HE.

## 2. Aim

The aim of the paper is to re-examine the latest diagnostic modalities, new information, and updated treatment strategies of HE.

## 3. Hepatic Encephalopathy

### 3.1. Pathophysiology

HE is an alteration of the central nervous system as a result of hepatic insufficiency, after the exclusion of other known metabolic, infectious, intracranial vascular events, or space-occupying lesions which may present itself with similar symptomology.

It manifests itself as a neuropsychiatric syndrome which may result in impairment of the sleep-wake cycle, cognition, memory, consciousness, personality changes, motor-sensory abnormalities, and decreased energy levels representing a continuum of manifestations of hepatic encephalopathy that can be experienced by the patient or elicited by the physician on history taking or physical examination [17].

Understanding the pathophysiology of a disease is important in developing effective treatment goals. The broad spectrum of neuropsychiatric manifestations reflects the range of pathophysiological mechanisms, for example, impairment of neurotransmitter systems, impaired cerebral perfusion and cerebral edema, and/or atrophy that interplay with one another. The challenge remains to dissect these purported mechanisms singularly for possible pharmacological intervention to improve treatment.

One of the major tenets of the pathophysiology of HE that remains firmly regarded is hyperammonemia that results from an increased nitrogenous load from the gastrointestinal tract. Ammonia is produced both by bacterial degradation of amines, aminoacids, purines, and urea as well as enterocytic glutaminase activity that converts glutamine to glutamate and ammonia.

In liver failure and cirrhosis, a diminished mass of functioning hepatocytes results in a decreased amount of ammonia being detoxified in the liver by conversion to urea via the Krebs-Henseleit cycle. In addition, portosystemic shunting also diverts a higher ammonia load to the systemic circulation by bypassing the portal system. The skeletal muscles and the kidneys compensate in ammonia metabolism with increased glutamine synthetase activity. However, astrocytes, which play a pivotal role in regulating the blood-brain barrier and neuronal homeostasis and nutrition, are not able to increase its glutamine synthetase activity to cope with the increased ammonia load [18, 19].

Neurotoxic agents such as ammonia gain entry into the brain and cause morphologic changes to the astrocytes (morphologic change to Alzheimer type 2 astrocytosis characterized by cell swelling), which leads to cerebral edema, raised intracranial pressure and brain herniation [20]. The true incidence of elevated intracranial pressure in cirrhosis and HE remains undetermined, although the presence of low grade cerebral edema in these groups of patients has been reported [21, 22].

Astrocyte swelling impairs its homeostatic ability and predisposes to neuronal dysfunction. It also induces oxidative stress by the formation of reactive oxygen species (ROS) which then goes on to cause further astrocyte swelling [23]. Görg B and colleagues affirmed the idea of oxidative and nitrosative stress in the pathogenesis of cerebral ammonia toxicity that have been alluded to in cell culture studies and animal models. They demonstrated elevated levels of protein tyrosine-nitrated proteins, heat shock protein-27, and 8-hydroxyguanosine in the cerebral cortex of HE patients with cirrhosis in a postmortem analysis of cortical brain tissue samples [24]. In addition, astrocyte swelling also leads to a depletion of taurine which has been associated with cerebral ammonia toxicity, although its role in HE pathophysiology is not fully recognized. Taurine has been reported to rescue hippocampal long-term potentiation from ammonia-induced impairment [25].

Astrocyte swelling is a result of intra-astrocytic accumulation of glutamine as a result of ammonia exposure. Intra-astrocytic glutamine has been accused in the “Trojan horse” hypothesis of mediating cerebral ammonia toxicity by increasing intracellular mitochondrial ammonia levels which in turns drives ROS production [26]. However, other factors such as hyponatraemia, inflammatory cytokines, and benzodiazepines have also shown to induce astrocyte swelling in vitro. All these may synergistically promote astrocyte swelling as a common pathogenetic endpoint [20, 21]. These factors may explain the presence of hepatic encephalopathy in patients with normal ammonia levels [27]. The postulate of increased apparent permeability-surface area (aPS) product in cirrhotic patients resulting in increased blood-brain permeability leaving the brain vulnerable to hyperammonemia may also be contributory to this phenomenon [28].

Impairment in neurotransmission involving changes in central neurotransmitters such as glutamate [29], catecholamines [30], serotonin [31], histamine [32], and melatonin [33] has been proposed and reported both in animal models and some in vivo studies, although none of these molecules have been proven sufficiently compelling to form the bedrock mechanism that underlies the development of HE. However, the increased inhibitory function of gamma-aminobutyric acid (GABA) as well as the upregulation of 18-kDa translocator protein (TSPO) (formerly referred to as the peripheral benzodiazepine receptor or PBTR) as a result of ammonia neurotoxicity is well known [34–36]. Upregulation of TSPO results in increased cholesterol uptake and synthesis of neurosteroids which have potent positive allosteric modulator properties on the GABA-A receptor system which could account for neural inhibition in HE [37–39]. In this respect, a Cochrane review found flumazenil led to a short-term improvement of HE in some patients with chronic liver disease and is a highly favorable prognosis [40]. Another meta-analysis by Goulenok and colleagues of six double-blind randomized controlled trials found flumazenil to induce clinical and electroencephalographic improvement over placebo of HE in patients with cirrhosis. [41] More recently, neurosteroids such as TGR5 (Gpbar-1) which is a membrane-bound bile acid receptor in the gastrointestinal tract and immune cells with pleiotropic actions, expressed also in astrocytes and neurons, has also been implicated as a novel neurosteroid receptor in the brain involved in the pathogenesis of HE [42]. Manganese has also been shown to be increased in patients with cirrhosis, and Krieger reported the association of increased manganese accumulation in the caudate nucleus, quadrigeminal plate, and globus pallidus with these increased levels which may have a role in HE [43].

### 3.2. Clinical Subtypes

A multiaxial definition and a type of nomenclature that characterizes both the type of hepatic abnormality and the duration/characteristics of the neurologic manifestations in chronic liver disease proposed by the Hepatic Encephalopathy Working Party at the 11th World Congresses of Gastroenterology in 1998 is briefly outlined below (Table 1).

Type A HE describes encephalopathy associated with acute liver failure. Type B HE describes encephalopathy associated with portal-systemic bypass and no intrinsic hepatocellular disease. Type C HE describes encephalopathy associated with cirrhosis and portal hypertension or portal-systemic shunts. Type C hepatic encephalopathy is, in turn, subcategorized as episodic, persistent, or minimal [17].

### 3.3. Diagnosis

According to the Hepatic Encephalopathy Working Group consensus statement, the diagnosis of HE should only be made after the exclusion of other brain disorders [17]. The diagnosis should be strongly considered in those patients who display disordered mentation or impairment of motor function, in the absence of metabolic and drug derangements, and a normal nervous system. A high degree of suspicion is required for patients with underlying liver cirrhosis in the presence of an acute precipitating event, and a careful neuropsychiatric evaluation must be performed.

Ammonia levels can be measured initially to support diagnosis and treatment in patients with a history of underlying liver cirrhosis. Venous samples of ammonia are adequate, and there does not appear to additional advantage in measuring partial pressures of ammonia [44]. However, it must be noted that the ammonia levels can be normal in 10% of patients in significant encephalopathy [27]. In addition, ammonia levels can be elevated in up to 69% of patients without signs and symptoms of encephalopathy [44].

The clinical utility of a single ammonia level in the diagnosis of HE is therefore uncertain given the substantial overlap of ammonia levels in both patients with and without encephalopathy. Other causes of hyperammonemia such as inappropriate blood draws or processing, drug-related causes such as sodium valproate, physiological factors such as high protein meals and strenuous exercise, and less commonly enzymatic deficiencies in urea cycle should be considered in a patient who does not have underlying cirrhosis or clinical encephalopathy.

Although elevated ammonia levels correlate with the severity of HE, reduced ammonia levels have shown to decrease encephalopathic symptoms [44], and repeated ammonia levels should not replace the clinical evaluation of patients as the relationship between ammonia levels and risk of cerebral edema in cirrhosis still remains to be evaluated [16].

Although there are several practical requirements for the diagnosis of minimal hepatic encephalopathy (MHE), there is no consensus on how it should be diagnosed [17, 45]. Several diagnostic methods such as formal neuropsychological assessment, short neuropsychological batteries, computerized tests (e.g., reaction time), and neurophysiological tests (critical flicker frequency, EEG, spectral EEG, and evoked potentials) have been proposed [45], but there is no ideal test for MHE as yet.

Specific batteries that have so far been recommended for assessment of neuropsychological disturbances associated with MHE include Portosystemic-Encephalopathy-(PSE) Syndrome Test [46], Psychometric-Hepatic-Encephalopathy-Sum (PHES)-Score, and the Repeatable Battery for the Assessment of Neuropsychological Status (RBANS) [47]. Meyer et al. utilized RBANS to evaluate neuropsychological changes in a large sample of liver transplant candidates [48], and RBANS has also been shown in a separate study to adequately characterize known patterns of cognitive dysfunction in patients with end-stage liver disease [49]. Such psychometric test batteries including PSE-Syndrome-Test and PHES-Score are, however, limited by retest reliability which may be influenced by several factors such as training and education. As such, normative values may vary across control sample size and may pose a hindrance in their utilisation in multicenterd studies. Kircheis and colleagues have also illustrated the limitations with PSE-Syndrome-Test and PHES-Score which includes the unreliable distinction between MHE and overt HE grade 1 in cirrhotic patients, although they have been shown to differentiate cirrhotic patients without signs of overt HE from controls [50–52]. This is attributed to the subjectivity of clinical assessment, problems in applying, and analyzing and scoring paper-pencil tests, but also owing to inconsistencies and the unbalanced contribution of single tests to the PHES-Score. It is suggested that the grading severity should be quantitatively measured by an objective physical and reproducible parameter for which neurophysiological tests may be an option [50].

In the local setting, we suspect the MHE is probably underrecognised and underdiagnozed. Since patients are diagnosed usually only by screening as they are relatively asymptomatic, a lack of awareness amongst physicians coupled with the prohibitively complicated and time consuming assessment and investigations, may contribute to its underdetection and underrecognised role in cirrhotic patients especially in a busy centre.

Some research has been done in identifying a serum biomarker for MHE. 3-Nitro-tyrosine has shown some early promise in detecting MHE, although this has to be validated further. This gives hope for a more simplistic approach to diagnosing MHE [53].

### 3.4. Staging

It is pertinent to assess the degree of HE. In this way, response to therapeutic interventions can be quantified and reproduced in a reliable fashion. Despite the numerous neuroimaging techniques available, clinical scales remain the best way to determine efficacy of response to therapy in HE.

The Glasgow Coma Scale has been referred to in the previous guidelines [16], but has not been specifically used and studied in HE, and is also used in other types of coma as well.

The West-Haven classification, formulated by Harold Conn and colleagues whilst investigating the therapeutic efficacy of lactulose, is a currently popular method in classifying the degree of HE [54] (Table 2).

#### 3.4.1. The West-Haven Classification Table

**Stage 1**

Minimal hepatic encephalopathy (previously known as subclinical hepatic encephalopathy). Lack of detectable changes in personality or behaviour. Minimal changes in memory, concentration, intellectual function, and coordination. Asterixis is absent.

**Stage 2**

Trivial lack of awareness. Shortened attention span. Impaired addition or subtraction. Hypersomnia, insomnia, or inversion of sleep pattern. Euphoria, depression, or irritability. Mild confusion. Slowing of ability to perform mental tasks. Asterixis can be detected.

**Stage 3**

Lethargy or apathy. Minimal disorientation. Inappropriate behaviour. Slurred speech. Obvious asterixis. Drowsiness, lethargy, gross deficits in ability to perform mental tasks, obvious personality changes, inappropriate behaviour, and intermittent disorientation, usually regarding time.

**Stage 4**

Somnolent but can be aroused, unable to perform mental tasks, gross disorientation about time and place, marked confusion, amnesia, occasional fits of rage, present but incomprehensible speech.

**Stage 5**

Coma with or without response to painful stimuli.

However, the terms that limit each stage of the classification are not clearly defined, and the metric characteristics of the stage are unknown. It is for this reason that other scales such as the Clinical Hepatic Encephalopathy Staging Scale (CHESS) have been proposed [55]. The presence or absence of the nine items on the CHESS score may be helpful in eliminating interobserver variability and in making a distinction between the various grades of encephalopathy. This staging scale, however, requires further validation.

## 4. Treatment Strategies

The treatment of HE includes excluding nonhepatic causes of altered mentation; treating precipitating factors, such as infection, dehydration, and electrolyte disturbances; initiating first-line therapy for hepatic encephalopathy; providing specialized nursing care with regular neurological assessments, and managing severe hepatic encephalopathy in the intensive care unit (ICU), if required.

Apart from identifying and treating underlying precipitants for each episode of HE, strategies to cope with hyperammonemia by reducing nitrogenous load in the gastrointestinal tract and counteracting its neurotoxic effects, antibiotic regimes, probiotics, liver dialysis using Molecular Adsorbent Recirculating System (MARS) and liver transplant help manage HE and ameliorate its consequence. It is, however, not in the scope of this paper to discuss the latter two modalities.

### 4.1. Targeting Hyperammonemia

One treatment target is to reduce the production and absorption of ammonia. To this end, dietary protein restriction is a mainstay in the management of acute HE amongst physicians since the 1950s [56]. This highly restrictive protein diet has possibly resulted in protein energy malnutrition in about 30–40% of cirrhotic patients by anthropometric data [57].

Newer data has since emerged to suggest that cirrhotic patients may have higher protein requirement than initially thought. In 1997, the European Society for Parenteral and Enteral Nutrition published consensus guidelines recommending that the daily protein intake in patients with liver disease should, if possible, be around 1.0–1.5 g/kg depending on the degree of hepatic decompensation [58].

Branch chain aminoacids (BCAAs) derived mainly from dairy products and vegetables account for about a quarter of total dietary protein, proving good substrates for conserving muscular mass in cirrhotic patients. BCAA deficiency may worsen glutaminergic neurotransmission and exacerbate protein-energy deficits. Whilst BCAAs were recommended to balance nitrogen balance, no clinical improvement in encephalopathy has been demonstrated [59, 60]. This has been confirmed recently, although BCAA supplementation shows some promise in improving MHE and muscle mass. In a randomised, double-blind, multicenter study involving 116 patients conducted by Les et al., The patients taking a supplement of 30 g of BCAA exhibited better outcome on 2 neuropsychological tests and an increase in midarm muscle circumference when compared to maltodextrin over 56 weeks [61].

In addition, cathartics, such as lactulose (beta-galactoside fructose) and lactilol (beta-galactoside sorbitol) are nonabsorbable disaccharides have long been considered standard treatment for hepatic encephalopathy [16]. It remains first line therapy in several institutions locally where we practice.

The American College of Gastroenterology practice guidelines released in 2001 recommend administering lactulose 45 mL/hour until evacuation occurs, then titrating the dose to two or three soft-stool bowel movements per day [16]. However, administration of such high volumes of lactulose is rarely practiced locally, and the use of stimulant or osmotic laxatives such as bisacodyl and sodium phosphate enemas is commonly used to augment the effects of lactulose.

Cathartics reduce intestinal ammonia load by its work as a cathartic and by acidification of the gut lumen which inhibits ammoniagenic coliform bacteria, leading to increased levels of nonammoniagenic lactobacilli. The acidification process results in retention of ammonia (NH_3_) in the colon as ammonium (NH_4_^+^), thus trapping it and preventing its absorption. The trapped ammonium is then expelled in the stool. Because of the potential ototoxic and nephrotoxic side effects of antibiotics such as neomycin and possible gastrointestinal and systemic side effects of metronidazole, lactulose is considered to be a safer alternative to antibiotics [62], although hypokalaemia and hypovolemia from severe overdosage may also actually trigger off encephalopathy and has to be administered judiciously.

Lactulose can be administered orally and rectally, although there is decreased time in lowering stool pH and serum ammonia levels when administered rectally [63]. Lactulose can be given via an enema, and the patient is to retain this for 30 to 60 minutes.

Whilst several small studies have validated the efficacy of lactulose [54, 64], a recent meta-analysis by Nielsen et al. contradicts these trials [65]. It was found that lactulose is no more effective than placebo in improving symptoms of encephalopathy and was actually inferior to antibiotic therapy. This meta-analysis forces reconsideration of the use of antibiotics, although a recent study by Sharma and colleagues showed that 11% (6/55) of patients with cirrhosis developed overt HE with lactulose compared to 28% (15/40) with placebo in an open-labeled, randomised, controlled trial studying primary prophylaxis of overt HE in cirrhotic patients [66].

In certain circumstances such as in the secondary prophylaxis of recurrent HE, the administration of lactulose has been demonstrated to be superior to placebo. Sharma BC and colleagues reported a reduction of HE episodes with lactulose (19.6%) versus placebo (46.8%) over a median followup of 14 months in 125 cirrhotic patients who were consecutively admitted for HE in a randomised placebo-controlled trial [67].

The overall transplant-free survival following a first episode of hepatic encephalopathy is only 42% at 1 year, and 23% at 3 years [1]. Gastrointestinal bleeding, in particular acute variceal bleed, is a common trigger of hepatic encephalopathy and lactulose has been demonstrated to be effective in preventing HE in patients with cirrhosis and acute variceal bleed. Sharma et al. examined the effectiveness of lactulose in prophylaxis of HE in acute variceal bleed. 70 patients presenting with acute variceal bleed without HE were randomised to receiving lactulose or no lactulose, with both groups receiving standard treatment for acute variceal bleed as per Baveno IV guidelines. 14% (5/35) in the lactulose group and 40% (14/35) in the non-lactulose group (*P* = 0.03) developed overt HE. In this study, the absolute risk reduction for HE was 26.2%, with lactulose, while the relative risk reduction was 65.5%. The number needed to prevent HE was 3.8 [68].

There may also be a role for lactulose in patients with MHE in preventing progression to overt encephalopathy and quality of life, although there was no significant difference in terms of mortality benefit [69].

### 4.2. Antibiotics

Antimicrobials reduce bacterial production of ammonia and other bacteria-derived toxins through suppression of intestinal flora. Antibiotics such as neomycin [54], vancomycin [70], metronidazole [71] and oral quinolones were previously shown in some studies to be efficacious in the treatment of both acute and/or chronic encephalopathy.

Although neomycin has been considered “standard” treatment for HE, the only randomised, placebo controlled study found no benefit of neomycin compared with standard treatment alone [72]. Also, the combination of neomycin with lactulose was not shown to be superior to placebo [73].

The newly approved FDA drug, Rifaximin has shown much promise. It is a minimally absorbed non-aminoglycoside bacteriacidal antimicrobial agent [74] with a broad spectrum in vitro and in vivo activity against gram-positive and gram-negative aerobic and anaerobic enteric bacteria [75]. There is suggestion that it compromises the physiologic function of pathogens in vivo, as well as inhibits the full expression of gene virulence and antibiotic resistance at sub-MICs (minimum inhibitory concentrations) [76].

The purported efficacy of Rifaximin in hepatic encephalopathy was first published in 1993 when it was compared to lactulose in patients with medium to severe encephalopathy [77]. It has since been demonstrated again to be superior to lactulose and antimicrobials in patients with mild-to-moderate severe HE [78] and appears a good option in reducing the risk of a breakthrough episode and hospitalisation involving HE.

Bass et al. compared Rifaximin at a dose of 550 mg twice daily versus placebo in a randomised, double-blind, placebo-controlled trial in 299 patients over a 6-month period. The risk of HE was reduced with Rifaximin with a hazard ratio of 0.42; 95% confidence interval (CI), 0.28 to 0.64; *P* < 0.001. In addition, risk of hospitalisation involving HE was lower in patients in the Rifaximin group, with a hazard ratio of 0.5; 95% CI, 0.29 to 0.87; *P* = 0.01 [79]. However, Rifaximin was not effective in a subgroup of patients who were not concurrently provided with lactulose and had increased scores (>18) on the model of End-Stage Liver Disease (MELD) scale [80]. One of the main limitations of the study was the lack of a real placebo group (i.e., receiving neither lactulose nor Rifaximin) since discontinuation of lactulose in patients at high risk for hepatic encephalopathy was deemed unethical. Hence, this limited the evaluation of the sole efficacy of Rifaximin [80, 81]. In addition, documentation of HE was carried out indirectly through perusal of case sheets and documents in 30–40% of cases. Given the high cost of treatment with Rifaximin and the current available data, it may be premature to conclusively recommend Rifaximin as first-line treatment in all cirrhotic patients.

### 4.3. Probiotics

Probiotics represent an attractive therapeutic option in the armamentarium of treatment strategies of HE. Considered, natural therapy, as occasionally considered as complementary medicine, it is relatively well tolerated even in cirrhotic patients [82].

The three suggested mechanisms through which probiotics exerts their efficacious effects and disrupt the pathogenesis of HE include the following [83]:

1. Decreasing total ammonia in the portal blood by
  - decreasing bacterial urease activity;
  - decreasing ammonia absorption by decreasing pH;
  - decreasing intestinal permeability;
  - improving nutritional status of gut epithelium.
2. Decreasing inflammation and oxidative stress in the hepatocytes
  - leading to increased hepatic clearance of ammonia and toxins.
3. Decreasing uptake of other toxins.

Probiotics have had encouraging results in improving psychometric test results and severity of liver disease (with improvement in the Child-Turcotte-Pugh score) in some studies [84–87]. Liu et al. screened 97 consecutive outpatients with cirrhosis but without overt HE and reported that the administration of synbiotics (probiotics and fermentable fibre) increased the fecal content of non-urease producing *Lactobacillus* species at the expense of other potentially pathogenic species such as *Escherichia coli* and *Staphylococcus * species. Such modulation of gut flora was associated with lowering of blood ammonia levels and reversal of MHE in 50% of patients [85].

However, a recent Cochrane meta-analysis revealed the lack of well-designed trials [88]. Larger followup studies are required to further validate the therapeutic potential of probiotics and to determine the sustained efficacy and tolerability before they can be recommended as standard treatment.

### 4.4. Other Potential Treatment Options

Flumazenil, a benzodiazepine antagonist, which targets the GABA receptor-complex pathway has shown modest benefit [40] in certain selected groups of patients such as those with severe hepatic coma [89] and encephalopathy associated with bleeding [90]. The restoration of central dopaminergic function seen in rats after administration of flumazenil is purported to be a contributing factor in the improvement of HE [91].

L-ornithine L-aspartate (LOLA) and Acetyl L-carnitine (ALC) have made some headway in demonstrating reduction in serum ammonia levels and improved mental function [92, 93]. There were also improvements to serum prothrombin time, serum bilirubin, and fasting ammonia levels in patients that were randomised to ALC in a randomized, double-blind, and placebo-controlled study administering ALC in cirrhotic patients [93]. However, there are concerns about the possibly transient ammonia lowering effects of LOLA with rebound hyperammonemia on cessation of the drug [94].

Sodium benzoate increases renal excretion of ammonia and has shown to be as effective as lactulose and cheaper alternative in acute portosystemic encephalopathy [95] and in the treatment of inborn errors of urea synthesis [96].

Acarbose can be considered in patients with low-grade HE and concomitant diabetes mellitus requiring oral hypoglycemic agents. This alpha-1 glucosidase inhibitor inhibits intestinal glucose absorption and thereby reducing substrate for ammonia production [97].

However, these therapies need further validation in a wider population and larger trials and cannot as yet be considered the first-line standard treatments for HE.

## 5. Conclusion

With the emergence of newer evidence, the 2001 American practice guidelines for HE needs to be reviewed to mirror updated treatment algorithms and paradigms to direct healthcare professionals in the evidence-based treatment of HE [98].

There has been a proposed treatment algorithm for HE [99] and an updated modified treatment algorithm for cirrhotic patients with MHE thus far [100]. However, there is a general lack of strong conclusive evidence to guide current practices [101]. A standardized approach to the treatment of persistent, episodic, recurrent, and minimal encephalopathy should be studied and proposed to reflect current contemporary therapeutic strategies and to benchmark future therapeutics in the field of HE.

Conventional wisdom which subscribes to lactulose and neomycin as standard treatment for HE has to be reexamined in the light of newer evidences. Rifaximin may turn out to be a suitable candidate for first-line treatment in the future, although its long-term effects still need to be assessed. Crucially, factors other than ammonia that contributes to the pathogenesis of the varied clinical spectrum of HE need to be identified.

To continue to push the frontiers of medical advancement in the field of hepatic encephalopathy, more robust and well-designed, randomized, controlled trials need to be designed to confirm and validate the efficacy of promising novel therapeutic approaches such as probiotics, sodium benzoate, L-ornithine L-aspartate (LOLA), and acetyl L-carnitine (ALC) as well as neuromodulators like flumazenil.

We currently recommend that in addition to treating acute precipitating causes of HE due to various causes, lactulose can currently still be considered for first-line therapy in the treatment of HE until further evidence emerges. Given the high cost of treatment with Rifaximin and the current available data, it may be premature to conclusively recommend Rifaximin as first-line treatment in all cirrhotic patients at this juncture.

## Figures and Tables

**Table 1:** 

Type	Description	Subcategory	Subdivision
A	Encephalopathy associated with acute liver failure	—	—
B	Encephalopathy associated with portal-systemic bypass and no intrinsic hepatocellular disease	—	—
C	Encephalopathy associated with cirrhosis and portal hypertension or portal-systemic shunts	Episodic	Precipitated
			Spontaneous
			Recurrent
		Persistent	Mild
			Severe
			Treatment-dependent
		Minimal	

**Table 2:** West-Haven criteria for hepatic encephalopathy.

Stage	Consciousness	Intellect and behavior	Neurological findings
0	Normal	Normal	Normal examination; if impaired psychomotor testing, consider MHE
1	Mild lack of awareness	Shortened attention span	Impaired addition or subtraction Mild asterixis or tremor
2	Lethargic	Disoriented; inappropriate behaviour	Obvious asterixis; slurred speech
3	Somnolent but arousable	Gross disorientation; bizarre behaviour	Muscular rigidity and clonus; hyperreflexia
4	Coma	Coma	Decerebrate posturing
